# Genkwadaphnin inhibits growth and invasion in hepatocellular carcinoma by blocking DHCR24-mediated cholesterol biosynthesis and lipid rafts formation

**DOI:** 10.1038/s41416-020-01085-z

**Published:** 2020-09-22

**Authors:** Jie Wu, Ling Guo, Xiaoran Qiu, Yong Ren, Feifei Li, Wei Cui, Shaojiang Song

**Affiliations:** 1 Key Laboratory of Computational Chemistry-Based Natural Antitumor Drug Research & Development, Liaoning Province, Shenyang Pharmaceutical University, Shenyang, 110016 People’s Republic of China; 2 School of Traditional Chinese Materia Medica, Shenyang Pharmaceutical University, Shenyang, 110016 People’s Republic of China; 3 School of Life Science and Biopharmaceutics, Shenyang Pharmaceutical University, Shenyang, 110016 People’s Republic of China; 4 Department of Pathology, Central Theater Command General Hospital PLA, Wuhan, Hubei 430070 People’s Republic of China

**Keywords:** Cancer therapy, Natural products

## Abstract

**Background:**

The liver is the central organ for cholesterol homoeostasis, and its dysfunction might cause liver pathological alterations including hepatocellular carcinomas (HCCs). 3β-hydroxysteroid-Δ24 reductase (DHCR24), a crucial enzyme of cholesterol biosynthetic pathway, is involved in lipid rafts formation. Genkwadaphnin (GD) is a daphnane diterpene isolated from the flower buds of *Daphne genkwa* Siebold et Zuccarini (Thymelaeaceae).

**Methods:**

We evaluated in vitro and in vivo effect of GD using HCC cells and BALB/c nude mice. Microarray assays were used to identify the differential genes by GD. DHCR24 expression and activity, cholesterol level, lipid rafts structure and the role of DHCR24 in human HCC specimens were tested by various molecular biology techniques.

**Results:**

High expression of DHCR24 in human HCC specimens was correlated with poor clinical outcome. Interfering DHCR24 altered growth and migration of HCC cells. GD inhibited growth and metastasis of HCC cells both in vivo and in vitro. GD suppressed DHCR24 expression and activity, as well as DHCR24-mediated cholesterol biosynthesis and lipid rafts formation, then further inhibited HCC cell invasion and migration.

**Conclusions:**

Our data suggest that DHCR24-mediated cholesterol metabolism might be an effective therapeutic strategy in HCC, and natural product GD might be a promising agent for HCC therapy.

## Background

Hepatocellular carcinoma (HCC) is predicted to be the fourth leading cause of cancer-related death worldwide.^1^ The 5-year survival rate for HCC patients is only 15%, and more than 80% of HCC patients are diagnosed at an advanced stage with poor prognosis.^2,3^ Most HCC patients cannot be cured by surgical treatment due to the rapid progression of the disease as well as widespread intrahepatic and extrahepatic metastasis.^4^ Increasing evidence indicates that hepato-carcinogenesis is not only induced by genetic and genomic alterations that ultimately lead to malignant transformation, but is also closely correlated with altered metabolism.^5^ However, the underlying mechanisms are incompletely understood. Clearly, there is an urgent need for better understanding of the molecular basis of HCC in order to develop effective treatment strategies to improve survival of patients with unresectable hepatic malignancies.

Recently, there has been great interest in the use of natural products with few adverse effects and low toxicity to treat various diseases.^6^ Genkwadaphnin (GD), a daphnane-type diterpene orthoester, is a natural product that we recently isolate from a traditional herbal medicine, namely the flower buds of *Daphne genkwa* Sieb. Et Zucc (Thymelaeaceae).^7–9^ A recent publication showed that the presence of daphnane-type diterpene orthoesters was limited to only a few plant families.^10^ These unique constituents possess a wide range of biological activities, including anti-tumour, cholesterol-lowering, anti-HIV, anti-fertility and anti-hyperglycaemic activities.^10^ In our previous study, we discovered that a series of daphnane-type diterpenes, including GD, exerted remarkable inhibitory effects on multiple cancer cell lines.^7,11^ GD has been reported to induce apoptosis in human myelocytic HL-60 cells and squamous cell carcinoma (SCC) cells, and to suppress tumour growth in mice inoculated with Lewis lung carcinoma (LLC) cells.^8,12^ Additionally, GD increases the expression of the tumour suppressor PRDM1 in human SW620 colon cancer cells.^9^ GD is also reported to be involved in the activation of PKD1 and ERK/MEK pathways, which activates NF-kB, thereby triggering IFN-γ production in NK-92 cells.^13^ However, the function and mechanism of GD in HCC cells are yet to be identified. Here, HCC cells Hep3B, PLC/PRF/5 and BALB/c nude mice with subcutaneous xenografts and tail vein injection lung metastasis models were used to investigate the mechanism of GD.

Several lines of clinical and experimental evidence have suggested the close relationship between liver cancer incidence and metabolic alterations, including cholesterol metabolism.^5,14,15^ These metabolic alterations provide the nutrients and energy to support the uncontrolled growth of malignant cells.^14,16^ Increased cholesterol levels have been reported to be positively associated with a higher incidence of cancers such as prostate, glioblastoma, bladder, colon and hepatic cancer.^17–20^ Lipid rafts are small, specialised cholesterol- and sphingolipid-enriched lipid domains within the cell membrane, which play a role in cellular signal transduction.^21^ Changes in membrane cholesterol-rich lipid rafts have been shown to affect cancer progression.^22–25^ Studies showed that disruption of lipid rafts domains by methyl-β-cyclodextrin (MβCD, a type of cholesterol chelator) inhibited the invasion and survival of several cancer cells.^15,26–28^ Although the effects of cholesterol-lowering drugs, statins, are controversial on some cancer, statins exhibit beneficial effects by reducing the risk and mortality in several types of cancers, including prostatic, gastric, oesophageal, and hepatic cancer, colorectal cancer, lung cancer and breast cancer.^28–31^ Therefore, targeting cholesterol metabolism, including cholesterol synthesis, might be a current strategy for anticancer therapy.^32,33^ 3β-Hydroxysteroid-Δ24 reductase (DHCR24, also called 24-dehydrocholesterol reductase, seladin-1), a pivotal enzyme of the cholesterol biosynthetic pathway, has been implicated in diverse biological processes.^34,35^ The overexpression of DHCR24 is associated with invasiveness and disease recurrence in multiple cancers.^36,37^ However, the regulatory mechanisms and biological behaviours of DHCR24 in HCC are still not fully elucidated. Therefore, we evaluate the biological function of DHCR24 in HCC and we identify GD as an effective natural inhibitor of DHCR24-induced cholesterol synthesis.

In the present study, we evaluated the in vitro and in vivo effect of GD using HCC cells and BALB/c nude mice. We found that GD was an effective anti-HCC agent by suppressing DHCR24-mediated cholesterol biosynthesis and lipid rafts formation, and by inhibiting HCC cells growth and metastasis. Additionally, we revealed that high expression of DHCR24 in HCC patients was markedly correlated with poor clinical outcome. We further demonstrated that DHCR24 was involved in cells invasion and migration by modulating cholesterol biosynthesis and lipid rafts formation in HCC. Taken together, these results imply that alteration of DHCR24-mediated cholesterol metabolism might be an effective therapeutic strategy in HCC, and GD has potential as a novel anticancer agent.

## Methods

### Compounds

Genkwadaphnin (GD), pimelotide A, yuanhuafine, yuanhuadine, genkwadane E, yuanhuapine, and yuanhualine with a purity more than 98% were isolated from an ethanol extract of the flower buds of *Daphne genkwa* as described previously.^7^ Lovastatin was purchased from Melone Pharmaceutical Company (Dalian, China).

### Cell culture

Hep3B and PLC/PRF/5 (human hepatoma cells), TE-1 (human oesophageal cancer cells), MCF-7 (human breast cancer cells), A549 (human lung carcinoma cells), U251 (human glioma cells), HL7702 (human normal liver cell) were cultured in Dulbecco’s modified Eagle’s medium (DMEM) with 10% foetal bovine serum (FBS) (Gibco BRL, Grand Island, USA) and 1% penicillin/streptomycin (100 U/mL penicillin, 100 μg/mL streptomycin) at 37 °C in a humidified atmosphere with 5% CO_2_. Cells were obtained from the American Type Culture Collection (Manassas, VA, USA). Cell lines has recently been authenticated by Guangzhou Aiji Biotechnology Co. LTD (Guangzhou, China).

### Cell viability

Cell viability was evaluated by MTT assay. After cells were treated with compounds for 48 h, 20 μL of MTT (Sigma, CA, USA) (5 mg/mL) was added for 4 h. Then 150 μL of DMSO/well was added to dissolve the crystals. The absorbance was measured at 490 nm using a microplate reader (Molecular Devices, Thermo, CA, USA).

### Preparation of peripheral blood mononuclear cells

All experiments and procedures were carried out according to the Regulations of Experimental Animal Administration issued by the State Committee of Science and Technology of China and approved by the Institutional Animal Care and Use Committee (permission number: SCXK (Liao) 2015-0001) of Shenyang Pharmaceutical University. 5 Male SD rats (250–280 g) aged 6 weeks old were purchased from Liaoning Changsheng Biotechnology Co., Ltd (Liaoning, China). The rats were housed in specific pathogen-free (SPF)-class housing cages containing wood shavings and bedding with controlled temperature (20–22°C), humidity (40 –60%), lighting (12 h light/dark cycle) and fed with sterile water and fodder. To ensure the health status of animals, rat weight and water and fodder consumption were monitored for a week. To collect blood, rats were euthanised using carbon dioxide inhalation in their home cages, followed by taking the blood from the heart of the 5 rats immediately. Fresh blood collected from 5 rats, respectively was then separated using sterile conical centrifuge tubes. 3 mL of Ficoll-Hypaque solution was slowly layered underneath the 10 mL of blood/PBS mixture by placing the solution at the bottom of the sample tube. After centrifugation (400 × *g*, 20 min), the peripheral blood mononuclear cells were harvested and washed in PBS (250 × *g*, 10 min) to remove platelets. The peripheral blood mononuclear cells were resuspended in DMEM (10% NBCS), and the cells were counted and incubated in 96-well culture clusters, then subjected to MTT assay (*n* = 5).

### Colony formation

Approximately 1000 cells/well were seeded into 6-well plates cultured for 12 days with indicated treatments to allow colony formation. The cells were fixed with 4% paraformaldehyde and stained with crystal violet. Then we counted colonies containing more than 50 cells.

### Wound-healing assay

Cells were seeded in 24-well plates until confluent, and a single scratch was made in the cell layer using a sterile 10 μl pipette tip. The cells were subsequently incubated with different concentrations of GD. Images of the scratches were captured at 48 h with an Olympus inverted microscope at ×100 magnification, and the width of each scratch was analysed.

### Transwell invasion assay

Invasion studies were performed using Transwell chambers (Millipore, Bedford, MA, USA). Matrigel (BD Biosciences, Bedford, MA, USA) was applied to the top side of an 8 μm pore polycarbonate filter. 8 × 10^4^ cells in serum-free medium were added into the upper chamber and the lower chamber was filled with DMEM containing 10% FBS. The cells on the lower surface were fixed and stained with crystal violet, then photomicrographed using Olympus microscope (Tokyo, Japan).

### Microarrays

Total RNA from Hep3B cells was extracted using TRIzol Reagent (Invitrogen, Shanghai, China). Microarray gene expression analysis was performed by Affymetrix Inc. (Santa Clara, CA, USA). Heatmaps were generated using Mev software and the log ratio of the red to green intensities for each signal was used for statistical analysis. Kyoto Encyclopedia of Genes and Genomes (KEGG) and gene ontology (GO) enrichment analyses were carried out to determine the top significant terms and pathways associated with the differentially expressed genes.

### Enzyme-linked immunosorbent assay (ELISA)

The enzymatic activity of DHCR24 was detected using Human DHCR24 ELISA kits (Zeye Biotechnology Co., Ltd, Shanghai, China) according to the manufacturer’s instructions. Briefly, 50 μl of each standard and sample were added to appropriate wells and incubated for 0.5 h at 37 °C with gentle shaking. After washing, 50 μl of Streptavidin-horseradish peroxidase (HRP) solution was added to each well and incubated for 30 min at 37 °C. Following another wash, 100 μl of 3,3′,5,5′-tetramethylbenzidine (TMB) substrate was added and incubated for 10 min at 37 °C in the dark with gentle shaking, followed by the addition of 50 μl stop solution. Standard curves were created.

### Total cholesterol assay

The levels of cholesterol were assayed using Tissue Total Cholesterol Assay kits (Applygen Technologies, Beijing, China) as described previously.^38,39^ All experiments were performed according to the manufacturer’s recommended protocol.

### SiRNA-mediated knockdown of gene expression

Cells were transfected with DHCR24 siRNAs (GenePharma, Shanghai, China) when the fusion level reached 70–80% using Lipofectamine 2000 reagent (Invitrogen, Shanghai, China) according to the manufacturer’s instructions. The siRNA sequences were described in Table 1.

**Table 1:** The siRNA sequences are as follows.

Name	Sense	Antisense
Negative control	UUCUCCGAACGUGUCACGUTT	ACGUGACACGUUCGGAGAATT
DHCR24-siRNA-1	GCUGAAUAGCAUUGGCAAUTT	AUUGCCAAUGCUAUUCAGCTT
DHCR24-siRNA-2	GCCGCGUGUGAAACACUUUTT	AAAGUGUUUCACACGCGGCTT

### Plasmid constructs for overexpression

Cells were seeded and transfected at 70–80% confluency with DHCR24 (untagged)-Human plasmid (#SC320135) (Origene, Beijing, China) using Lipofectamine 2000 reagent (Invitrogen, Shanghai, China). After the indicated treatments, transfected cells were harvested for analysis.

### Quantitative real-time PCR assay

The samples were collected for total RNA isolation with Trizol (Invitrogen, Carlsbad, CA, USA) and Ultrapure RNA Kit (Cwbio, Beijing, China) according to the manufacturer’s instructions. Reverse transcription of mRNA to template cDNA was performed using PrimeScript RT Master Mix (TaKaRa, Beijing, China). cDNA was amplified with gene-specific primers and the synthesised cDNA was analysed by quantitative real-time PCR using SYBR Green Permix Ex Taq ROX plus (TaKaRa, Beijing, China). Gene expression data were normalised to the housekeeping gene β-actin, and the relative abundance of mRNA transcripts was calculated as 2^−ΔΔCT^ method with Mx3000P Real-Time PCR Detection System (Agilent, CA, USA). The primer sequences used in the assay were summarised in Table 2.

**Table 2:** The primer sequences used in the quantitative real-time PCR experiments.

Gene	Forward primer 5′–3′	Reverse primer 5′–3′
DHCR24	GCACAGGCATCGAGTCATCAT	GTGCATCGCACAAAGCTGC
β-Actin	TTGCGTTACACCCTTTCTTG	CACCTTCACCGTTCCAGTTT
PAK1	AGGGGAGTTTACGGGAATGC	TCTTCTGCTCCGACTTAGTGATA
NR2F2	TCATGGGTATCGAGAACATTTGC	TTCAACACAAACAGCTCGCTC
FGFR1	GGCTACAAGGTCCGTTATGCC	GATGCTGCCGTACTCATTCTC
MMP16	AGCACTGGAAGACGGTTGG	CTCCGTTCCGCAGACTGTA
HMGCR	CAAGGAGCATGCAAAGATAATCC	GCCATTACGGTCCCACACA
CYP51A	GCAGGGATGCTTATTGGATTACTC	AGAAGCCCATCCAAGCACTAG
SQLE	GAGATGGAAGAAAGGTGACAGTCA	CACCCGGCTGCAGGAAT
DHCR7	ACGCTACACTCTGCCCACACT	CAAGAGTAAATGCAGCCTAATGACA

### Western blot assay

Samples that after treatment were harvested and lysed in RIPA buffer on ice supplemented with 1 mM PMFS to obtain total cellular protein. Cells lysates were centrifuged at 14,000 × *g* for 15 min at 4 °C and protein concentrations were determined using a BCA protein assay kit (Thermo Fisher Scientific Co., Ltd Shanghai, China). Cell extracts were denatured at 100 °C for 10 min in loading buffer and equivalent amounts of protein (40 μg) were separated on 10% SDS-PAGE gels. Separated protein bands were transferred onto PVDF membranes (0.22 μm, Millipore) using an electroblotting apparatus (Bio-Rad, CA, USA) and the membranes were blocked in 5% skim milk powder in TBST for 1 h. Blots were subsequently incubated with primary antibodies overnight at 4 °C followed by the corresponding horseradish peroxidase (HRP)-conjugated secondary antibody for 2 h. The immune-reactive bands were detected using ECL reagents and Molecular Imager ChemiDoc XRS + System (Bio-Rad, CA, USA). Antibody information was summarised in Table 3.

**Table 3:** The antibodies used in the experiments.

Antibody	Companies purchased from	Dilution
DHCR24	Cell Signaling Technology (#2033)	WB: 1:1000; IHC: 1:200
β-Actin	Proteintech (#20536-1-AP)	WB: 1:1000
E-Cadherin	Cell Signaling Technology (#9782)	WB: 1:500
Vimentin	Cell Signaling Technology (#9782)	WB: 1:500
MMP9	Proteintech (#10375-2-AP)	WB: 1:500
MMP2	Proteintech (#10373-2-AP)	WB: 1:500
Ki67	Abcam (#ab92742)	IHC: 1:500

### Immunofluorescence assay

The samples were stained with Cholera Toxin Subunit B (CT-B) Conjugates (Thermo Fisher Scientific, Shanghai, China) according to the manufacturer’s recommended protocol as described previously.^40,41^ Cells on cover slips were fixed and permeabilised. After non-specific antigen-binding sites blocking, the lipid rafts (green) and nuclei (blue) were stained by Alexa Fluor 488-conjugated CT-B and DAPI, respectively. Cells labelled with distinct bright green patches on the plasma membrane were counted which indicated lipid rafts^+^ cells. Immunofluorescent images were obtained under a confocal fluorescence microscopy (Nikon C2, Tokyo, Japan).

### In vivo anti-tumour activity and metastasis assays

All experiments and procedures were carried out according to the Regulations of Experimental Animal Administration issued by the State Committee of Science and Technology of China and approved by the Institutional Animal Care and Use Committee (permission number: SCXK (Jing) 2014-0004) of Shenyang Pharmaceutical University. Male BALB/c nude mice at the age of 6 weeks (18–20 g) were purchased from Beijing HFK Bioscience Co., Ltd (Beijing, China) and housed with controlled temperature (20−22 °C), humidity (40−60%), lighting (12 h light/dark cycle) and sterile water and fodder in SPF-class housing cages containing wood shavings and bedding. For subcutaneous xenografts experiments to evaluate the anti-tumour effect of GD, 1 × 10^6^ Hep3B cells were injected into the right backsides of each BALB/c nude mouse. One week later, when the tumour size reached about 100 mm^3^, the mice were randomly divided into 3 groups (*n* = 4/group): Group 1 Control; Group 2 GD (1 mg/kg); Group 3 GD (2 mg/kg). GD was dissolved in 0.5% carboxymethyl cellulose sodium (CMC-Na) with deionised water and given orally each day for 18 days. The tumour volume (V) was determined by measuring the length (L) and width (W) with a digital calliper and calculated according to the formula: V (mm^3^) = 0.5236 × L × W^2^. At the same time, the weight of each mouse was measured every day. To ensure the health status of animals, fodder and water consumption were monitored. At the end of the treatment period, tumour-bearing mice were euthanised using carbon dioxide inhalation in their home cages. The tumours, livers, spleens and kidneys were excised immediately and fixed with 4% paraformaldehyde for immunochemical studies to evaluate the anti-tumour activity and toxicity of GD.

For tail vein injections experiments to evaluate the anti-tumour metastasis of GD, a total of 5 × 10^5^ PLC/PRF/5 cells were intravenously (i. v.) injected into the tail vein of BALB/c nude mice in 100 μL PBS to induce the pulmonary experimental metastasis model. Approximately 7 days following injection, the mice were treated with vehicle (CMC-Na) or GD (1 mg/kg, 2 mg/kg) orally for 10 days. At the end of the treatment period, tumour-bearing mice were euthanised using carbon dioxide inhalation in their home cages. Then, we evaluated the results after GD treatment. Lung metastasis quantification was performed by intratracheally injecting 5 mL of 15% Ink into each lung, then fixing and staining the lungs overnight in Fekete’s solution.^42^ Tumour nodules were enumerated using a dissecting microscope.

### H&E staining and immunohistochemistry

Tumours and mouse organs (liver, spleen, kidney and lung) were isolated and fixed in formalin and embedded in paraffin. The tissues were cut into 4-μm-thick sections, dewaxed, and stained with haematoxylin and eosin (H&E). Four-micrometre-thick tissue sections and TAM were prepared from the paraffin-embedded tissues. The sections were immune-stained and detected by using the streptavidin-perosidase method (MXB Biotech, Fuzhou, China). The slides were lightly counterstained with haematoxylin, and staining was evaluated with light microscope (Olympus, Tokyo, Japan). Antibody information was summarised in Table 3.

### Clinical and histological evaluation of human tissues

A total of 43 surgical resection specimens of HCC patients were obtained in Central Theater Command General Hospital PLA (Wuhan, PR, China). And a paraffin-embedded tissue microarray containing 90 cases of HCC specimens was obtained from Outdo Biotech Ltd. (Shanghai, China). Ethical oversight and approval were obtained from the Institutional Review Board of Wuhan General Hospital of Guangzhou Command. The clinicopathologic features of these patients have been summarised in Supplementary Table S1.

### Statistical analyses

All experiments were repeated at least three times and the in vitro data were presented as mean ± SD. Results in vivo were expressed as mean ± SEM. Statistical comparisons among the different groups were made using Student’s *t*-test and ANOVA in Graph Pad Prism (Version 7.0, GraphPad Software). *P*-values < 0.05 were considered statistically significant.

## Results

### GD reduces the viability of hepatocellular carcinoma cells

**Fig. 1:**
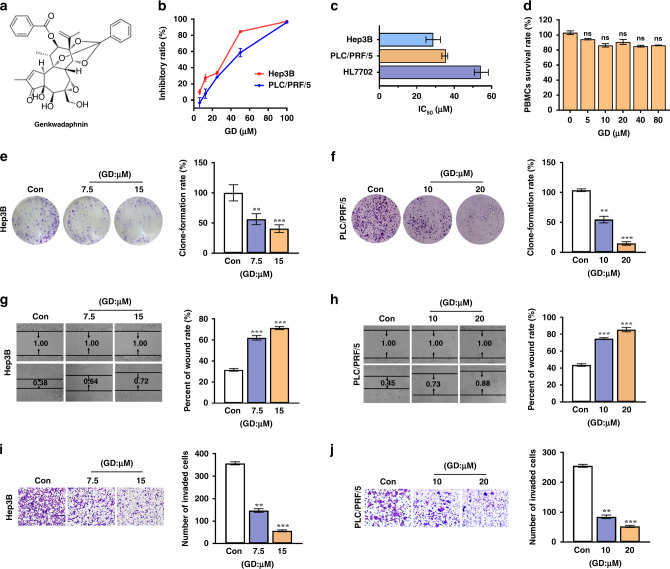
GD represses hepatocellular carcinoma cells growth, migration and invasion. **a** Chemical structure of GD. **b** Cells were exposed to GD at the indicated doses for 48 h and dose-escalation effects of GD was assessed by MTT assay. **c** The inhibitory intensities were expressed as IC_50_. **d** Viability of PBMCs obtained from rats after exposure to GD at various concentration for 48 h (*n* = 5). **e**, **f** The colony formation ability of HCC cells with different concentration of GD treatment for 12 days. **g**, **h** Cell migration was estimated by wound-healing assay. Cells were seeded in 24-well plates with GD at the indicated doses for 48 h. Quantifications were shown on right. **i**, **j** Cell invasion ability was estimated with Transwell chambers. Representative microscopy images (×20) of invasive cells with different concentration of GD treatment for 48 h. Quantifications were shown on right. *n* = 3, means ± SD, *P < 0.05, **P < 0.01, ***P < 0.001 compared with control group.

Our previous studies showed that several daphnane-type diterpenes isolated from flower buds of *Daphne genkwa* had significant cytotoxic activities.^7,11^ Here, we evaluated the in vitro cytotoxic potency of 7 daphnane-type diterpenes (Fig. 1a and Supplementary Fig. S1) against 6 human cancer cell lines including TE-1, MCF-7, A549, U251 Hep3B, PLC/PRF/5 cells by MTT assay with 5-fluorouracil (5-FU) as the positive control (Supplementary Table S2). Among all the compounds analysed, GD displayed the most potent cytotoxicity against multiple cell lines (Supplementary Table S2). Further results showed that GD markedly reduced the viability of human hepatoma Hep3B and PLC/PRF/5 cells in a concentration-dependent manner (Fig. 1b). Also, GD had a relatively weak cytotoxic effect on the normal human liver cell line HL7702, based on IC_50_ calculations (Fig. 1c). In order to further evaluate the cytotoxicity of GD, rat peripheral blood mononuclear cells (PBMCs) were prepared and treated with different doses of GD (*n* = 5). We found no obvious differences on the viability of PBMCs with increased GD concentration (Fig. 1d). In addition, the colony formation assay was performed to investigate the chronic growth inhibition of GD on single HCC cell. The numbers of colonies formed by HCC cells were dramatically reduced by GD in a dose-dependent manner (Fig. 1e, f). Collectively, these results indicate that GD has a strong anti-proliferative effect on HCC cells, with less toxicity to normal cells in vitro.

### GD suppresses the migration and invasion of hepatocellular carcinoma cells

Reduced cell adhesion and increased migratory ability are the most prominent features of cancer cells.^43^ These properties, which are related to increased metastasis, are the major causes of poor prognosis and cancer death.^43^ To determine whether GD had any effect on cell migration and invasion, we first conducted wound-healing assay and discovered that the gaps between the cells were bigger with GD treatment than that in the control group (Fig. 1g, h). Next, we examined the effect of GD on the cell invasion with Transwell assay. The results showed that GD significantly reduced the number of invading cells (Fig. 1i, j). Altogether, these data imply that GD suppresses HCC cells migration and invasion.

### GD treatment reduces the expression and activity of DHCR24

**Fig. 2:**
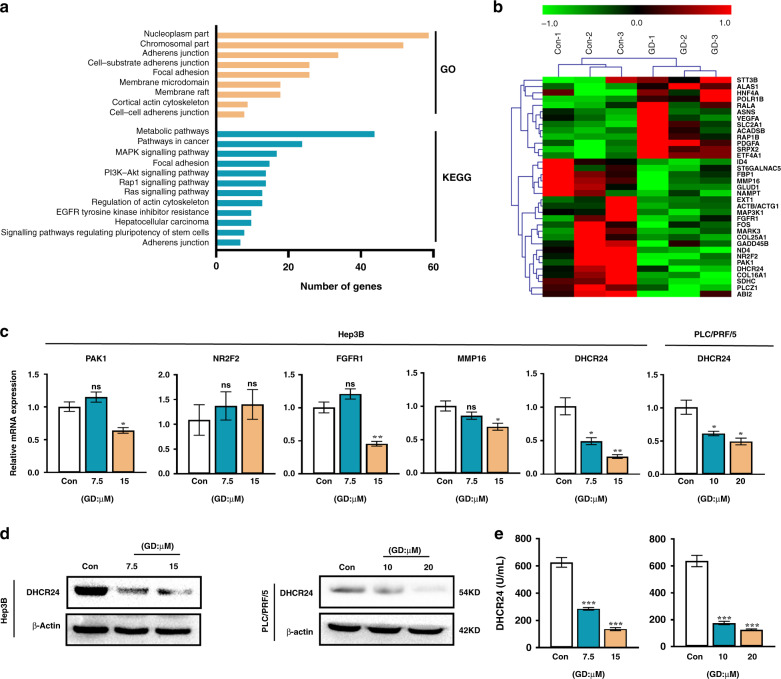
Differential gene profiling of Hep3B cells with GD treatment. GD represses DHCR24 expression and activity. **a** Hep3B cells were treated with 7.5 μM GD in triplicate for 48 h and the global gene expression change in the GD treatment group and corresponding control group was analysed by microarray assay. GO enrichment analysis (cellular component) and KEGG pathway analysis of the differentially expressed protein-coding genes affected by GD. **b** Heatmaps exhibit significant differential genes involved in metabolism pathway and the pathways identified in KEGG and GO analyses. **c** Validation of some differential expressed genes by quantitative real-time PCR analysis in Hep3B and PLC/PRF/5 cells treated with GD at the indicated doses for 48 h. **d** The effect of GD on DHCR24 protein expression was determined by western blot analysis. Whole-cell lysates were analysed by antibodies against DHCR24 protein with β-actin as loading control. **e** ELISA analysis of DHCR24 enzyme activity in Hep3B and PLC/PRF/5 cell lines with indicated concentrations of GD treatment. *n* = 3, means ± SD, *P < 0.05, **P < 0.01, ***P < 0.001 compared with control group.

In order to understand the anti-tumour mechanisms of GD, we conduct microarray assays to identify global gene expression changes in GD-treated human hepatoma Hep3B cells compared to vehicle-treated (control) cells. As expected, there were significant differences in the global transcription profile between the two sets of samples (*n* = 3 for each). Out of the genes screened, 219 genes were differentially expressed upon GD treatment, with 69 genes upregulated and 150 genes downregulated (Supplementary Fig. S2). Using the differentially expressed mRNAs as the input, we identified many enriched pathways by KEGG analysis, among which metabolic pathways and several metastasis-related pathways were the most significant (Fig. 2a). We also analysed the differentially expressed genes using GO analysis. We discovered that the list of enriched GO terms included “membrane microdomain” and “membrane raft”, although they were not the most significant (Fig. 2a). We then conducted clustering analysis of the differentially expressed genes which were involved in metabolic pathways and the pathways identified in the KEGG and GO analyses (Fig. 2b). Among these, there are 35 markedly changed genes which related to metabolic pathways and the pathways identified in the KEGG and GO analyses, we chose 5 (*PAK1*, *NR2F2*, *FGFR1*, *MMP16* and *DHCR24*) for verification by quantitative real-time PCR analysis. The results demonstrated that, among the detected genes, *DHCR24* was the most dramatically downregulated by 48 h of GD treatment in a dose-dependent manner in Hep3B and PLC/PRF/5 cell lines (Fig. 2c). Recent evidence has demonstrated that DHCR24 is highly associated with the cholesterol metabolism pathway and membrane lipid rafts.^35^ These results are consistent with the microarray results, so Hep3B and PLC/PRF/5 cell lines are selected to further test the inhibitory effects of GD on DHCR24 expression. Both western blot and ELISA assay results showed that GD inhibited the expression and enzyme activity of DHCR24 in HCC cells (Fig. 2d, e). Together, these results suggest that DHCR24 might play an important role in the anti-tumour effects of GD.

### DHCR24 knockdown suppresses cholesterol biosynthesis and lipid rafts formation, leading to reduce cell growth, migration and invasion

**Fig. 3:**
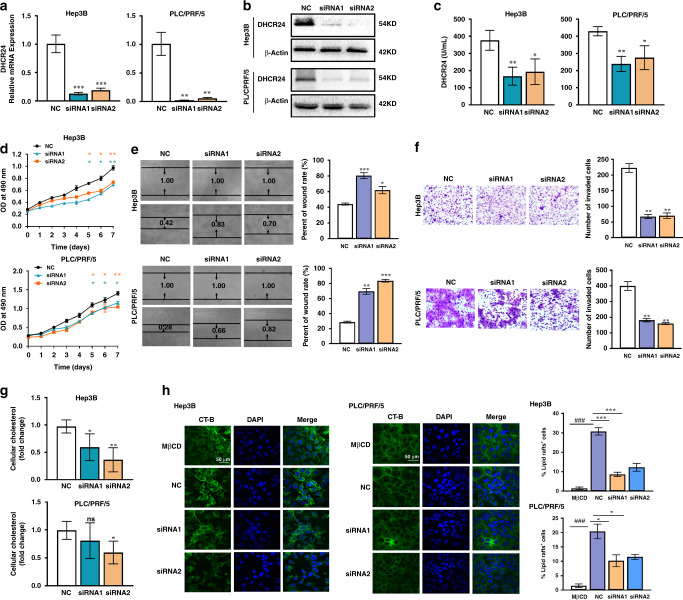
Knockdown of DHCR24 suppresses cholesterol biosynthesis and lipid rafts formation, leading to a reduction of cell growth, migration and invasion. The efficiency of DHCR24 knockdown was evaluated by quantitative real-time PCR (**a**) and western blot (**b**) analyses in Hep3B and PLC/PRF/5 cells. **c** DHCR24 activity was assessed using the DHCR24 ELISA assay kit after the knockdown of DHCR24. **d** MTT assay results indicated the effects of DHCR24 siRNA on cell growth. Effects of DHCR24 knockdown on migration (**e**) and invasion (**f**) of Hep3B and PLC/PRF/5 cells were detected by wound-healing assay and Transwell invasion assay. Quantifications were shown on right. **g** Cholesterol levels in Hep3B and PLC/PRF/5 cells was measured by tissue total cholesterol assay kit after the silencing of DHCR24. **h** Immunofluorescent staining of lipid rafts in Hep3B and PLC/PRF/5 cells after transfection with DHCR24 siRNA for 48 h, MβCD was used as positive control. Green signalling represents lipid rafts staining and blue indicates nuclei stained with DAPI. Scale bar, 50 μm. Data are representative of three experiments with similar results. means ± SD, *P < 0.05, **P < 0.01, ***P < 0.001 compared with control group. ^#^*P* < 0.05, ^##^*P* < 0.01, ^###^*P* < 0.01 MβCD group compared with control group.

Next, we use siRNA-mediated knockdown to investigate the exact role of DHCR24 in HCC cells. Transfection of HCC cells with siRNAs against DHCR24 resulted in a significantly reduced expression level of DHCR24 compared to control cells, as judged by quantitative real-time PCR (Fig. 3a) and western blot assays (Fig. 3b). Furthermore, we demonstrated a moderate and significant decrease of DHCR24 enzyme activity in HCC cells with DHCR24 knockdown measured by ELISA assay (Fig. 3c). As shown in Fig. 3d, OD_490_ value was significantly decreased after transfection DHCR24 siRNA1 and siRNA2 compared with the scramble control. The results indicated that siRNA-mediated knockdown impaired the growth in Hep3B and PLC/PRF/5 cells. We then conducted wound-healing and Transwell assays and declared that the migration and invasion of HCC cells were significantly decreased after DHCR24 knockdown, which indicated that DHCR24 might play an important role in HCC metastasis (Fig. 3e, f). As a vital enzyme of the cholesterol biosynthetic pathway, DHCR24 has been recently linked to regulation of cholesterol content and lipid rafts.^35^ Therefore, we measured tissue total cholesterol and discovered that DHCR24 knockdown led to a significant decrease of cholesterol levels in HCC cells (Fig. 3g). Next, we examined the distribution of the lipid rafts biomarker CT-B in HCC cells. We used methyl-β-cyclodextrin (MβCD), which depleted membrane cholesterol and acted as a lipid rafts disruptor, as the positive control. Immunofluorescence staining results showed that MβCD strongly reduced CT-B signal. Correspondingly, CT-B signal was significantly downregulated after siRNA-mediated DHCR24 knockdown in HCC cells (Fig. 3h). Altogether, the results suggest that interfering with the activity of DHCR24 downregulates cholesterol biosynthesis and lipid rafts formation, as well as inhibits the growth and metastasis of HCC cells.

### GD counteracts the DHCR24-overexpression-induced cholesterol biosynthesis and lipid rafts formation, and inhibits the growth, invasion and migration of HCC cells

**Fig. 4:**
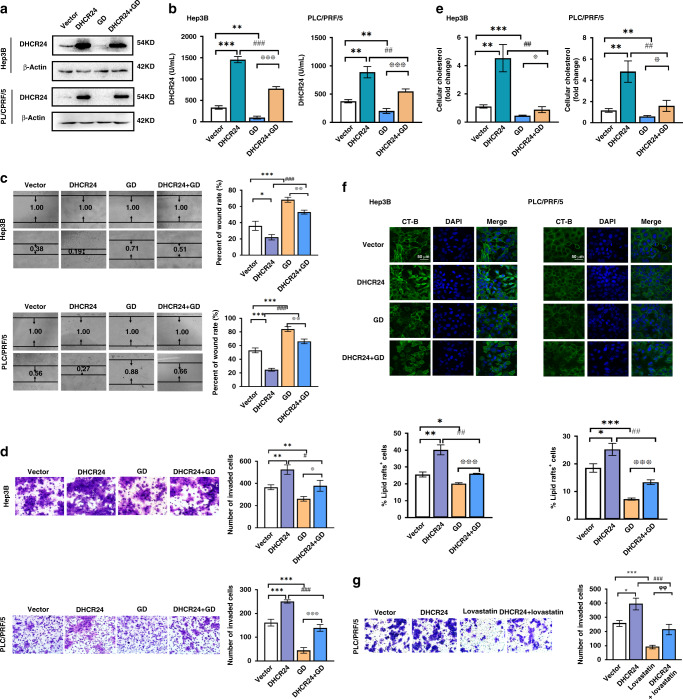
GD counteracts DHCR24-overexpression-induced cholesterol biosynthesis and lipid rafts formation and inhibits cell migration and invasion. Hep3B and PLC/PRF/5 cells were transfected with DHCR24 plasmid, followed by stimulation with or without GD for 48 h. **a** The expression levels of DHCR24 proteins were detected by western blot analysis. **b** DHCR24 activity was assessed using the DHCR24 ELISA assay kit. Migration (**c**) and invasion (**d**) of Hep3B and PLC/PRF/5 cells were detected by wound-healing assay and Transwell invasion assay. Quantifications were shown on right. **e** Cholesterol levels in Hep3B and PLC/PRF/5 cells was measured by tissue total cholesterol assay kit. **f** Immunofluorescent staining of lipid rafts in Hep3B and PLC/PRF/5 cells. Green signalling represents lipid rafts biomarker CT-B and blue signalling indicates nuclei stained with DAPI. Scale bar, 50 μm. **g** PLC/PRF/5 cells were transfected with DHCR24 plasmid, followed by stimulation with or without lovastatin for 48 h. Invasion of PLC/PRF/5 cells was detected by Transwell invasion assay. Quantifications were shown on right. *n* = 3, means ± SD, *P < 0.05, **P < 0.01, ***P < 0.001 versus control; ^#^*P* < 0.05, ^##^*P* < 0.01, ^###^*P* < 0.01 compared to DHCR24 overexpression group; ^※^*P* < 0.05, ^※※^*P* < 0.01, ^※※※^*P* < 0.01 compared to GD group; ^φ^*P* < 0.05^, φφ^*P* < 0.01, ^φφφ^*P* < 0.01 compared to lovastatin group.

To further confirm the effect of DHCR24 in HCC cells and elucidate how GD regulates migration and invasion, we performed overexpression experiments by transfecting cells with DHCR24 plasmid and treating them with GD. We first performed western blot assay to confirm that cells transfected with DHCR24 plasmid had stronger expression of DHCR24 than cells transfected with empty vector (Fig. 4a). DHCR24-transfected cells treated with GD for 48 h had lower levels of DHCR24 expression than untreated DHCR24-transfected cells, which implied that GD downregulated DHCR24 expression (Fig. 4a). Accordingly, ELISA assays revealed that DHCR24 enzyme activity was upregulated after DHCR24 transfection, and this upregulation was significantly reduced by GD treatment (Fig. 4b). In order to detect the effect of GD on cells growth, we performed the cell viability assay in two groups after DHCR24 gene manipulation. The results displayed that knockdown of DHCR24 in Hep3B cells (DHCR24 High level) and then treated with 15 μM GD resulted in the decrease of cell viability, whereas overexpression of DHCR24 in PLC/PRF/5 cells (DHCR24 Low level) and then treated with 30 μM GD could enhance cell viability (Supplementary Fig. S3). The results suggest DHCR24 involved in the anti-growth effect of GD. Subsequently, wound healing and Transwell assays were conducted. The results demonstrated that DHCR24 overexpression significantly increased the migration and invasion of HCC cells (Fig. 4c, d), and these DHCR24-induced changes were blocked by GD (Fig. 4c, d). We also tested the cellular cholesterol levels and the expression of the lipid rafts biomarker CT-B in DHCR24-overexpressing HCC cells with or without GD treatment. We noticed a significant elevation of the cholesterol level in the DHCR24-overexpressing cells compared with the negative control group (Fig. 4e). However, the DHCR24-induced upregulation of cholesterol level was reversed with GD treatment (Fig. 4e). Immunofluorescence staining results revealed that overexpression of DHCR24 upregulated CT-B signal (Fig. 4f). However, the elevation was blocked by GD treatment (Fig. 4f). Collectively, the results indicate that GD blocks the growth and migration of HCC cells by disrupting DHCR24-mediated cholesterol biosynthesis and formation of membrane lipid rafts. These findings support the potential therapeutic use of cholesterol biosynthesis inhibitors to suppress the growth and migration of cancer cells.

In order to confirm cholesterol’s effect on growth and invasion of HCC cells, we used a cholesterol-depleting agent, lovastatin, in DHCR24-overexpressing HCC cells to detect the cholesterol level, cells growth, migration and invasion upon lovastatin treatment. Lovastatin decreased the cholesterol level in Hep3B and PLC/PRF/5 cell lines (Supplementary Fig. S4a). Furthermore, overexpression of DHCR24 increased cholesterol level was impaired by lovastatin treatment (Supplementary Fig. S4a). Subsequently, the effect of lovastatin on cells growth was detected by MTT assay. The results displayed that the inhibitory effect of lovastatin on cells growth was attenuated in DHCR24 overexpression HCC cells (Supplementary Fig. S4b). Then, wound healing and Transwell assays showed that overexpression of DHCR24 accelerated migration and invasion in HCC cells (Supplementary Fig. S4c and Fig. 4g). Lovastatin blocked wound healing and cell invasion rates in HCC cell lines (Supplementary Fig. S4c and Fig. 4g). Meanwhile, in DHCR24 plasmid-transfected HCC cells, wound healing and cell invasion rates were still suppressed with lovastatin treatment (Supplementary Fig. S4c and Fig. 4g). The results demonstrate that cholesterol depletion drug, lovastatin, suppresses cells growth, migration and invasion, which suggests that DHCR24-induced cholesterol biosynthesis involved in growth and invasion of HCC cells.

### GD inhibits growth and metastasis of HCC cells in vivo

**Fig. 5:**
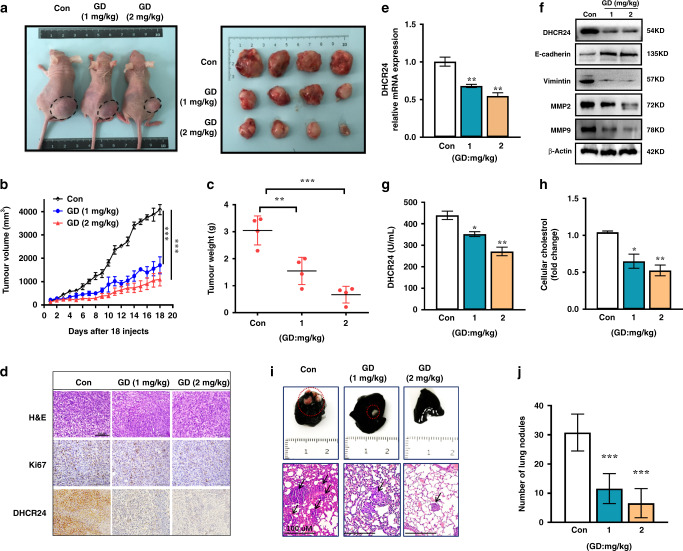
GD inhibits growth and metastasis of HCC cells in vivo. GD has therapeutic efficacy in Hep3B-bearing xenograft mice. **a** Representative image of tumour-bearing mice 18 days after indicated concentration of GD administration. **b** Volume of Hep3B subcutaneous tumours was determined at different time points. **c** Tumour weight of each group at the end of treatment. **d** The representative haematoxylin and eosin (H&E) staining of Hep3B xenograft tumour tissues. Immunohistochemical staining for Ki67 and DHCR24. **e** Tumour tissue lysates were subjected to quantitative real-time PCR assay to measure the expression of the target proteins DHCR24. **f** Western blot was carried out to evaluate the DHCR24 expression and EMT marker E-cadherin, Vimentin, MMP2 and MMP9 expression. **g** Quantitative ELISA analysis of DHCR24 activity in Hep3B xenograft tumour tissues was assessed using the DHCR24 ELISA assay kit. **h** Tissue total cholesterol assay kit was used to detect the cholesterol levels in Hep3B bearing xenograft tumour tissues with different groups. **i** The representative lung tissue and H&E staining of lung sections with tumour nodules. **j** The numbers of lung nodules. Data are presented as the means ± SEM and confirmed by three or more independent experiments. Statistical analysis was performed with Student’s *t*-test, *P < 0.05, **P < 0.01, ***P < 0.001 versus vehicle control group.

In order to evaluate whether the biological effect of GD is potentially clinically relevant, we test the in vivo effect of GD on tumour growth in BALB/c nude mice with Hep3B xenografts. After 1 week of acclimatisation, the mice grow well. When Hep3B tumours reached an average volume of 100 mm^3^, the mice were treated with vehicle (CMC-Na) or GD orally at 1 or 2 mg/kg/d for 18 days. To ensure the health status of animals, we monitored the mice weight and fodder and water consumption every day and found no obvious adverse effects. As shown in Fig. 5a, b, GD significantly inhibited the growth of the xenograft tumours. The tumour volumes in the 1 mg/kg GD-treated group were approximately 40% of those in the vehicle-treated mice at the end of treatment. And 2 mg/kg GD treatment resulted in an approximately 70% decrease in tumour volume compared with the vehicle control group. In addition, analysis of tumour weight revealed that GD-treated mice had much smaller tumour masses (Fig. 5c). Further histopathological studies showed that there were no obvious differences in the liver, kidney and spleen between the GD-treated groups and the vehicle control group, as judged by microscopic examination of tissue sections (Supplementary Fig. S5). Collectively, these results demonstrate that GD obviously inhibits tumour growth and has a good safety profile.

To further clarify the mechanism by which GD inhibited tumour growth in vivo, we analysed the expression levels of Ki-67 and DHCR24 in tumour samples by immunohistochemical staining. The fraction of Ki-67-positive proliferating cells and DHCR24 level were reduced with GD administration (Fig. 5d). Western blot and quantitative real-time PCR assays further demonstrated that expression of DHCR24 was strongly attenuated at the mRNA and protein levels within tumours from GD-treated mice (Fig. 5e, f). In addition, matrix metalloprotein-2 (MMP2) and matrix metalloprotein-9 (MMP9) protein expression were decreased by GD treatment in a dose-dependent manner (Fig. 5f). Moreover, western blot results demonstrated an increased protein expression of E-cadherin and a decreased protein expression of Vimentin within tumours from GD-treated mice (Fig. 5f). These results implied that tumour metastasis was suppressed with GD treatment in vivo. More importantly, ELISA analysis showed that DHCR24 enzyme activity was strongly decreased in xenograft tumour samples from GD-treated animals (Fig. 5g). Finally, we demonstrated a significantly reduced cholesterol level in tumours from mice treated with GD as compared with the control group (Fig. 5h). Taken together, these data indicate that GD exhibits anti-tumour activity in vivo, which is associated with the suppression of DHCR24-induced cholesterol biosynthesis.

On the basis of the above findings that GD changes the expression of EMT marker in subcutaneous xenograft tumours experiment and inhibits cell migration and invasion in vitro, we, therefore, investigated the effect of GD on HCC metastatic ability in vivo. To establish cancer model in vivo, PLC/PRF/5 cells were injected into tail vein of male nude mice. We observed number of lung metastatic nodules was reduced with GD administration as compared with that in control group (Fig. 5i, j). These results further verified the effect of GD on tumour metastasis.

### High expression of DHCR24 in HCC human specimens is correlated with poor clinical outcome

**Fig. 6:**
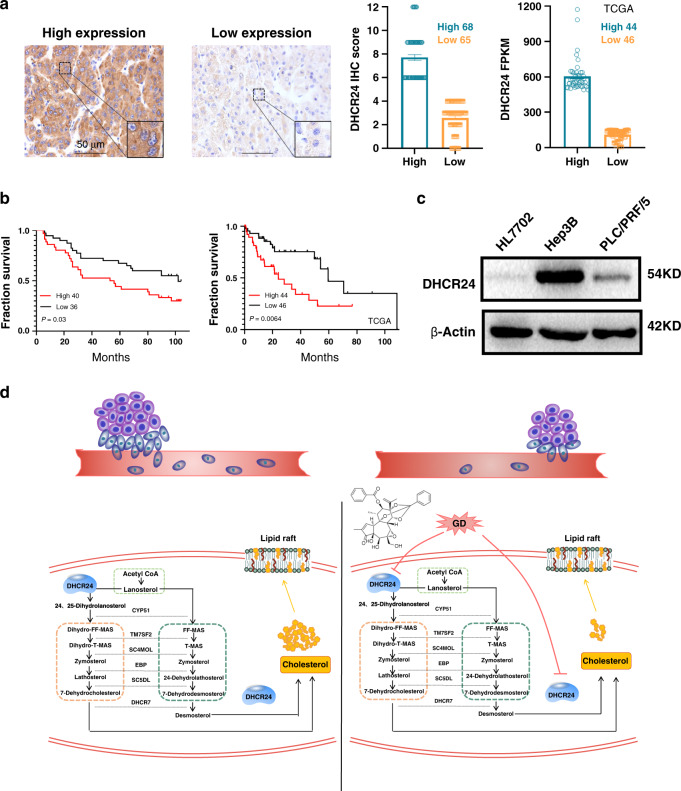
DHCR24 overexpression in HCC patients is correlated with poor clinical outcome. **a** Representative immunohistochemical staining for DHCR24 in human HCC specimens. IHC score of expression DHCR24 in low (*n* = 65) and high (*n* = 68) groups of 133 cases. Gene expression levels of DHCR24 in low (*n* = 46) and high (*n* = 44) risk groups of liver cancer patients from TCGA. **b** Kaplan–Meier analysis of the surgical resection specimens of HCC patients estimated the overall survival of HCC patients with DHCR24 different expression. Left side DHCR24 expression in low (*n* = 36) and high (*n* = 40) risk groups of HCC patients. Right side the HCC Dataset from TCGA estimated the overall survival of HCC patients with DHCR24 gene alteration. *P* < 0.05 DHCR24 in low group compared with DHCR24 in high group. **c** The basic expression level of DHCR24 protein in these hepatoma cell lines including Hep3B, PLC/PRF/5 and one normal liver cell line HL7702 was evaluated by western blot analysis. **d** Schematic diagram summarising the working model that GD inhibits growth and invasion in hepatocellular carcinoma by blocking DHCR24-mediated cholesterol biosynthesis and lipid rafts formation. DHCR24 is a vital enzyme of cholesterol synthesis from desmosterol to cholesterol. Enzymatic reactions and enzymes involved: CYP51 (C14-demethylase), TM7SF2 (D14-reductase), SC4MOL (C4-demethylase), EBP (D8D7-isomerase), SC5DL (D5-desaturase), DHCR7 (D7-reductase).

Until now, few studies have reported the expression of DHCR24 in HCC patient tissues. Here, a total of 133 specimens of HCC patients in two separately cohort (cohort1, 90 cases and cohort2, 43 cases) were detected (Fig. 6a and Table S1). Figure 6a right showed the IHC expression levels of DHCR24 in 133 cases (high group *n* = 68, low group *n* = 65), and the gene expression levels of DHCR24 in low (*n* = 46) and high (*n* = 44) risk groups of HCC patients from TCGA. Then we conducted Kaplan–Meier analysis to estimate the overall survival of HCC patients in Cohort1, we found that high expression of DHCR24 in HCC human cases was correlated with poor clinical outcome (Fig. 6b). In consistent with Cohort1 results, the increased expression of DHCR24 also predicted a poor prognosis in Cohort2 (Supplementary Fig. S6). Meanwhile, the HCC dataset from TCGA also showed that overexpression of DHCR24 in 44 HCC patients was correlated with short survival (Fig. 6b).

Furthermore, we determined the expression level of DHCR24 by western blot assay in two human HCC cell lines and one normal liver cell line (Fig. 6c). The results demonstrated that DHCR24 protein level was markedly increased in HCC cells compared with HL7702 (Fig. 6c). From these results, we conclude that DHCR24 is overexpressed in human HCC specimens and HCC cells, and high expression of DHCR24 in HCC specimens is correlated with poor clinical outcome.

## Discussion

Several epidemiological studies have shown that targeting lipid metabolism, including cholesterol metabolism, is a realistic strategy for treating aggressive cancers, because cancer cells have distinct metabolic requirements in order to accommodate their rapid proliferation.^5,15,44^ Cholesterol is an integral component of cellular membranes and plays an essential role in maintaining their integrity and function.^17,45,46^ The cholesterol-enriched membrane microdomains known as lipid rafts contain a large number of cancer-related signalling and adhesion molecules, and have been implicated in various cellular functions.^23,28^ Therefore, removing cholesterol or blocking cholesterol synthesis impairs several cellular signalling transduction events mediated by lipid rafts, and thus prevents uncontrolled cell proliferation and metastasis.^27,30^ Although our understanding of the metabolic alterations that drive cancer progression has improved, there is still a lack of effective therapeutic strategies. In this study, we conducted microarray assays to identify global gene expression changes in Hep3B cells upon GD treatment. KEGG analysis of the significantly altered genes uncovered that metabolic pathways were affected by GD treatment. GO analysis results revealed that GD treatment led to enrichment of the terms “membrane microdomain” and “membrane raft”. Additionally, we found that GD had an anti-HCC effect by suppressing DHCR24-mediated cholesterol biosynthesis and lipid rafts formation, and then inhibiting HCC cell growth and invasion. Meanwhile, inhibitor of cholesterol biosynthesis, lovastatin, was used in DHCR24-overexpressing HCC cells to further confirm cholesterol’s effect on growth and invasion of HCC cells. All the results suggested that the depletion of cholesterol might be a useful strategy for HCC prevention or treatment.

Currently, various inhibitors of metabolic enzymes are under clinical trial as targeted cancer therapeutics. The cholesterol is synthesised entirely from acetate through a complex series of over 30 enzymatic reactions. Two intersecting pathways have been described for the biosynthesis of cholesterol from lanosterol: the Bloch and Kandutsch–Russell pathways.^35,47,48^ These pathways are parallel, with the same sequence of reactions occurring, except the entry and the exit reaction. DHCR24 has been involved in the two pathways and termed as ‘lanosterol reductase’ or ‘desmosterol reductase’ depending on the sterol substrate (Fig. 6d). In the Kandutsch–Russell pathway, DHCR24 acts as ‘lanosterol reductase’ and the side chain is reduced early by DHCR24. Whereas, in the Bloch pathway, DHCR24 acts as ‘desmosterol reductase’ and the side chain is reduced as the final step by DHCR24. In addition, this enzyme DHCR24 can act upon any intermediate containing the C-24,25 double bond, intertwining the two pathways.^34,35,47,48^ Therefore, DHCR24 plays a pivotal role in the formation and stability of cholesterol. DHCR24 has been associated with multiple cellular functions, such as oxidative stress reduction, neuroprotection, anti-apoptotic function, and anti-inflammatory activity.^34,35^ Interestingly, the expression of DHCR24 is upregulated in various tumours.^36,37,49^ In the present study, we firstly discovered that the expression of DHCR24 was significantly downregulated in GD-treated Hep3B cells, as measured by microarray assays. Secondly, we further confirmed that DHCR24 was involved in the GD-induced anti-growth and anti-metastasis effects in Hep3B and PLC/PRF/5 cell lines. Additionally, some other crucial cholesterol synthesis-related enzymes, including *HMGCR*, *SQLE*, *CYP51A1* and *DHCR7*, couldn’t be significantly regulated in mRNA levels by GD, suggesting the unique role of DHCR24 in GD action (Supplementary Fig. S7). Meanwhile, recent reports demonstrated that in hepatitis C virus (HCV)-related HCC, overexpression of DHCR24 was induced by HCV, which indicated that DHCR24 could be a valuable therapeutic target for HCV-related HCC.^50,51^ In our study, we tested two HBV-related HCC cell lines and found that the expression of DHCR24 was upregulated in all of them compared to the normal liver cell line. We also discovered that higher DHCR24 expression was observed in patients with poor clinical outcome. Additionally, reduction of cholesterol-enriched lipid rafts by siRNA knockdown of DHCR24 was associated with significantly reduced migration and invasion in HCC cells. Correspondingly, upregulation of DHCR24 increased cholesterol biosynthesis and sustained lipid rafts construction, then accelerated the invasion and migration of HCC cells. More importantly, we found that GD suppressed DHCR24-mediated cholesterol biosynthesis and lipid rafts formation, and inhibited HCC cell growth, invasion and migration. The results are in line with previous reports showing the important role of DHCR24 in tumour growth and metastasis, which also predict that DHCR24 might become an effective target in HCC therapy.

Recently, bioactive natural products discovered in traditional herbal medicines have become important resources for new drug research and development, since they have relatively few side-effects compared to modern chemotherapeutics.^6^ Traditional herbal medicines are a complex combination of many natural products with rich structural diversity and promising therapeutic applications in various diseases.^6^ The flower buds of *Daphne genkwa* Sieb.et Zucc. (Thymelaeaceae) is a well-known traditional medicinal herb which has been used for thousands of years for the treatment of ascites, oedema, and asthma.^7,9^ The main ingredients of the genus Daphne include flavonoids, lignans, and daphnane-type diterpenes.^7,9^ Among these, daphnane-type diterpenes are unique constituents found in only a few plant families of Thymelaeaceae and Euphorbiaceae.^10^ They reportedly possess a wide variety of bioactivities, including anti-tumour, cholesterol-lowering, anti-fertility, anti-HIV, neurotrophic and anti-hyperglycaemic activities.^10^ Here, we evaluated the anti-cancer activity of some daphnane-type diterpenes from *Daphne genkwa*. GD exhibited the most significant anti-cancer activity, with less cytotoxicity to normal cells and no toxicity to PBMCs. Previous studies have shown that treatment with a daphnane-type diterpene resulted in the upregulation of p21 expression and growth arrest in cancer cell lines.^11^ GD induced apoptosis in human myelocytic HL-60 cells and suppressed tumour growth in mice inoculated with lung carcinoma cells.^12^ GD also induced reactive oxygen species (ROS)-mediated apoptosis of squamous cell carcinoma (SCC) cells.^8^ However, the function and mechanism of action of GD in HCC cells have not been explored. More importantly, we used in vivo and in vitro tests to demonstrate for the first time that GD inhibited the growth and invasion of HCC cells by regulating cholesterol metabolism and lipid rafts formation. Although more experimental methods and deeper mechanisms need to be studied, this discovery provides new ideas for the further use and development of this kind of compound and implies that GD might be a promising agent for HCC therapy.

In conclusion, we reveal that a natural product, GD, suppresses HCC growth and invasion by blocking DHCR24-induced cholesterol synthesis and lipid rafts formation (Fig. 6d). This study illustrates the crucial role of DHCR24 in the progression of HCC and leads to the prediction that cholesterol metabolism and lipid rafts formation will be important targets for HCC treatment and prevention. Importantly, our investigation provides new insights into the anti-cancer mechanism of daphnane-type diterpenes and suggests that the natural product GD might be valuable as a potential drug for cancer therapy.

## Supplementary information

Supplementary Files

## Data Availability

All data presented within the article and its Supplementary information files are available upon request from the corresponding author.
