# Supplementary material for: Genkwadaphnin inhibits growth and invasion in hepatocellular carcinoma by blocking DHCR24-mediated cholesterol biosynthesis and lipid rafts formation
Source: Br J Cancer. 2020 Sep 22;123(11):1673–85. doi: 10.1038/s41416-020-01085-z (PMC7686505; doi:10.1038/s41416-020-01085-z)
Supplement: Supplementary file 1 — Supplementary Files [file 41416_2020_1085_MOESM1_ESM.docx]

**Genkwadaphnin inhibits growth and invasion in** **hepatocellular carcinoma by blocking DHCR24-mediated** **cholesterol biosynthesis and lipid rafts formation**

Jie Wu ^1,2*^, Ling Guo ^3*^, Xiaoran Qiu^3^, Yong Ren ^4^, Feifei Li ^1,2^, Wei Cui ^3#^, Shaojiang Song ^1,2#^

**Affiliations:**

^1^ Key Laboratory of Computational Chemistry-Based Natural Antitumor Drug Research & Development, Liaoning Province, Shenyang Pharmaceutical University, Shenyang 110016, People's Republic of China

^2^ School of Traditional Chinese Materia Medica, Shenyang Pharmaceutical University, Shenyang 110016, People’s Republic of China

^3^ School of Life Science and Biopharmaceutics, Shenyang Pharmaceutical University, Shenyang 110016, People’s Republic of China

^4^ Department of Pathology, Central Theater Command General Hospital PLA, Wuhan, Hubei 430070, People’s Republic of China

*These authors contributed equally to this article.

**^#^Co-corresponding authors:**

Shaojiang Song, Key Laboratory of Computational Chemistry-Based Natural Antitumor Drug Research & Development; School of Traditional Chinese Materia Medica, Shenyang Pharmaceutical University, Shenyang 110016, Liaoning Province, China. Tel: (+86-24) 43520707; Fax: (+86-24) 43520707; E-mail: songsj99@163.com.

Wei Cui, School of Life Science and Biopharmaceutics, Shenyang Pharmaceutical University, Shenyang 110016, Liaoning Province, China. Tel: (+86-24) 23986265; Fax: (+86-24) 23986265; E-mail: [cuiwei_syphu@126.com](mailto:cuiwei_syphu@126.com)

**Supplementary Figure Legends**

**Fig. S1.** Chemical structure of the daphne diterpenes: pimelotide A, yuanhuafine, yuanhuadine, genkwadane E, yuanhuapine, yuanhualine.

**Fig. S2.** Heatmaps of gene-expression data from microarray analysis. Hep3B cells were treated with 7.5 μM GD in triplicate for 48h and the global gene-expression change in the treated group was normalized by corresponding control. Differentially expressed genes were determined by Student’s t test and a threshold cutoff of *p* < 0.05, 2-fold change. Red, induced; green, repressed; log2-base scale.

**Fig. S3.** Knockdown of DHCR24 in Hep3B cells (DHCR24 High level) resulted in the decrease of GD sensitivity, whereas overexpression of DHCR24 in PLC/PRF/5 cells (DHCR24 Low level) could enhance cell sensitivity to GD. Western blot showed DHCR24 protein expression indicating knockdown or overexpression.

**Fig. S4.** Cholesterol biosynthesis inhibitor, lovastatin, inhibits growth and migration of HCC cells. Hep3B and PLC/PRF/5 cells were transfected with DHCR24 plasmid, followed by stimulation with or without lovastatin for 48 hours. (a) Cholesterol levels in Hep3B and PLC/PRF/5 cells was measured by tissue total cholesterol assay kit. Growth (b) and migration (c) of Hep3B and PLC/PRF/5 cells were detected by MTT assay and wound-healing assay. Quantifications were shown on right. n=3, means ± SD, **P* < 0.05, ***P* < 0.01, ****P* < 0.001 versus control; ^#^*P* < 0.05, ^##^*P* < 0.01, ^###^*P* < 0.01 compared to DHCR24 overexpression group; ^φ^*P* < 0.05, ^φφ^*P* < 0.01, ^φφφ^*P* < 0.01 compared to lovastatin group.

**Fig. S5.** GD displays no obvious toxic damage on the liver, spleen and kidney in Hep3B-bearing xenograft mice. The representative staining histopathological examination of liver, spleen and kidney tissues of mice, as shown by H&E staining.

**Fig. S6.** The relationship between DHCR24 expression and survival in 40 cases of surgical resection patients.

**Fig. S7.** GD displays no obvious effects on some other crucial genes of cholesterol synthesis, such as *DHCR7*, *CYP51A1*, *HMGCR* and *SQLE* in Hep3B and PLC/PRF/5 cells.

**Supplementary Tables**

| **Table S1**. The clinicopathological parameters of 133 patients with HCC. | |
| --- | --- |
| **Variable** | **N** |
| **Age** |  |
| < 52 years | 43 |
| ≥ 52 years | 90 |
| **Gender** |  |
| Male | 115 |
| Female | 18 |
| **HBsAg** |  |
| Negtive | 28 |
| Positive | 105 |
| **Tumor size** |  |
| ≤5cm | 81 |
| >5cm | 52 |
| **Tumor differentiation** |  |
| I | 8 |
| II | 78 |
| III | 47 |
| **Tumor encapsulation** |  |
| Complete | 74 |
| None | 50 |
| **Vascular invasion** |  |
| No | 82 |
| Yes | 39 |

| **Table S2**. Inhibition effects of compounds on the growth of tumor cells *in vitro^a^*. | | | | | | |  |
| --- | --- | --- | --- | --- | --- | --- | --- |
| Compounds | IC50: *μ*M | | | | | |  |
|  | TE-1 | MCF-7 | Hep3B | A549 | U251 | PLC/RPF/5 |  |
| pimelotide A | 64.69±2.51 | 57.28±2.34 | 43.83±1.63 | 54.74±1.55 | 61.09±2.22 | 78.04±1.98 |  |
| yuanhuafine | 77.76±2.85 | >100 | 69.26±2.33 | 67.83±2.33 | >100 | >100 |  |
| yuanhuadine | 49.82±3.42 | 54.86±1.23 | 21.00±2.12 | 31.49±0.76 | 62.14±1.55 | 70.91±2.82 |  |
| genkwadane E | 65.79±3.95 | 58.95±1.87 | >100 | 45.56±1.23 | 56.99±1.94 | 67.33±1.56 |  |
| yuanhuapine | 69.67±0.96 | >100 | >100 | 65.72±1.65 | 58.58±1.87 | 56.34±2.56 |  |
| yuanhualine | 56.89±1.56 | 34.57±0.98 | 69.40±0.87 | 98.46±1.44 | 56.23±2.14 | 56.88±2.32 |  |
| genkwadaphnin | 27.05±2.16 | 23.39±1.91 | 31.24±2.11 | 23.03±2.64 | 54.37±2.96 | 37.81±0.91 |  |
| 5-FU | 33.23±3.45 | 15.58±2.78 | 40.34±1.77 | 44.05±2.11 | 22.11±1.67 | 48.21±1.45 |  |
| ^a^Results are expressed as IC_50_ values ± SD in *μ*M, 5-fluorouracil and DMSO were used as the positive and negative controls, respectively. The experiments were performed three times. | | | | | | |  |
|  |  |  |  |  |  |  |  |

**Supplementary Figures**

**
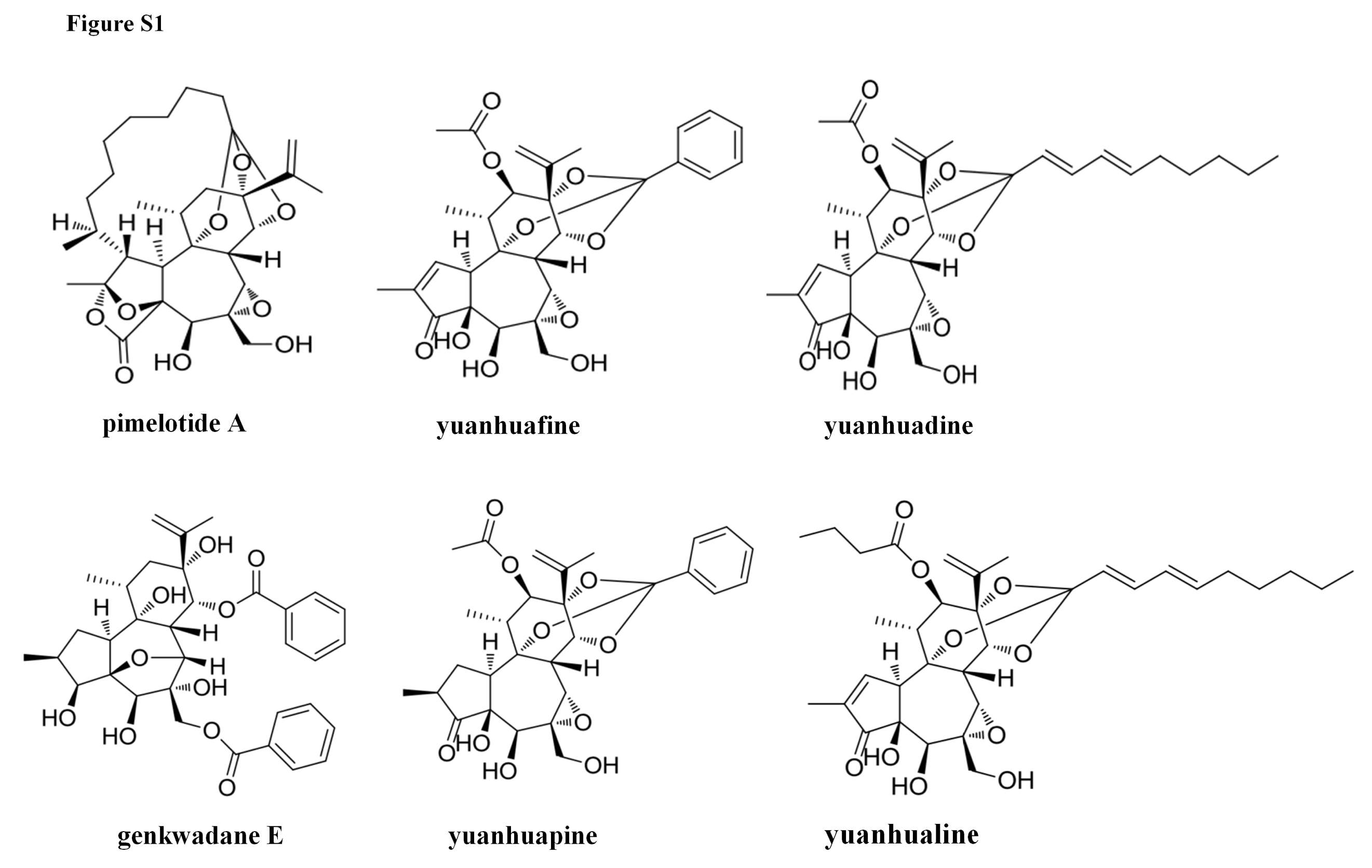
**

**
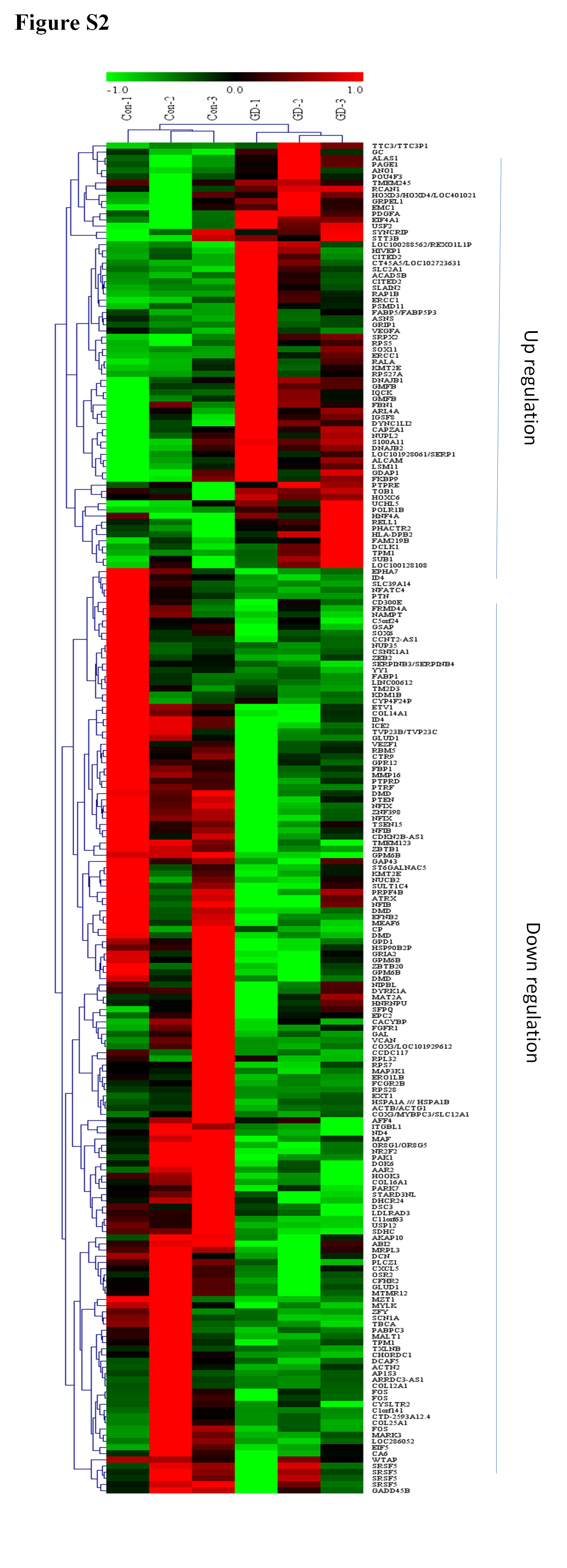
**

**
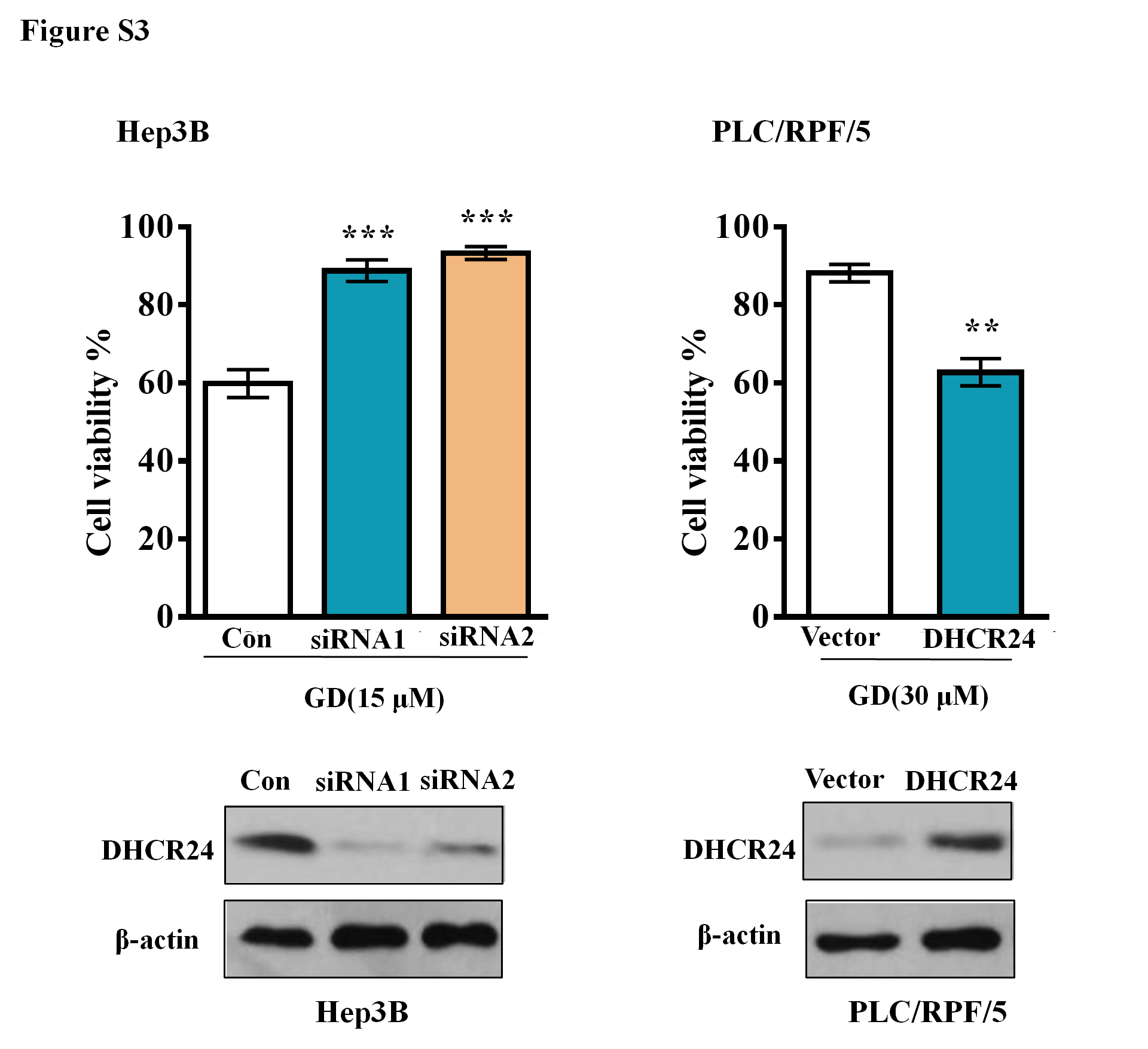
**

**
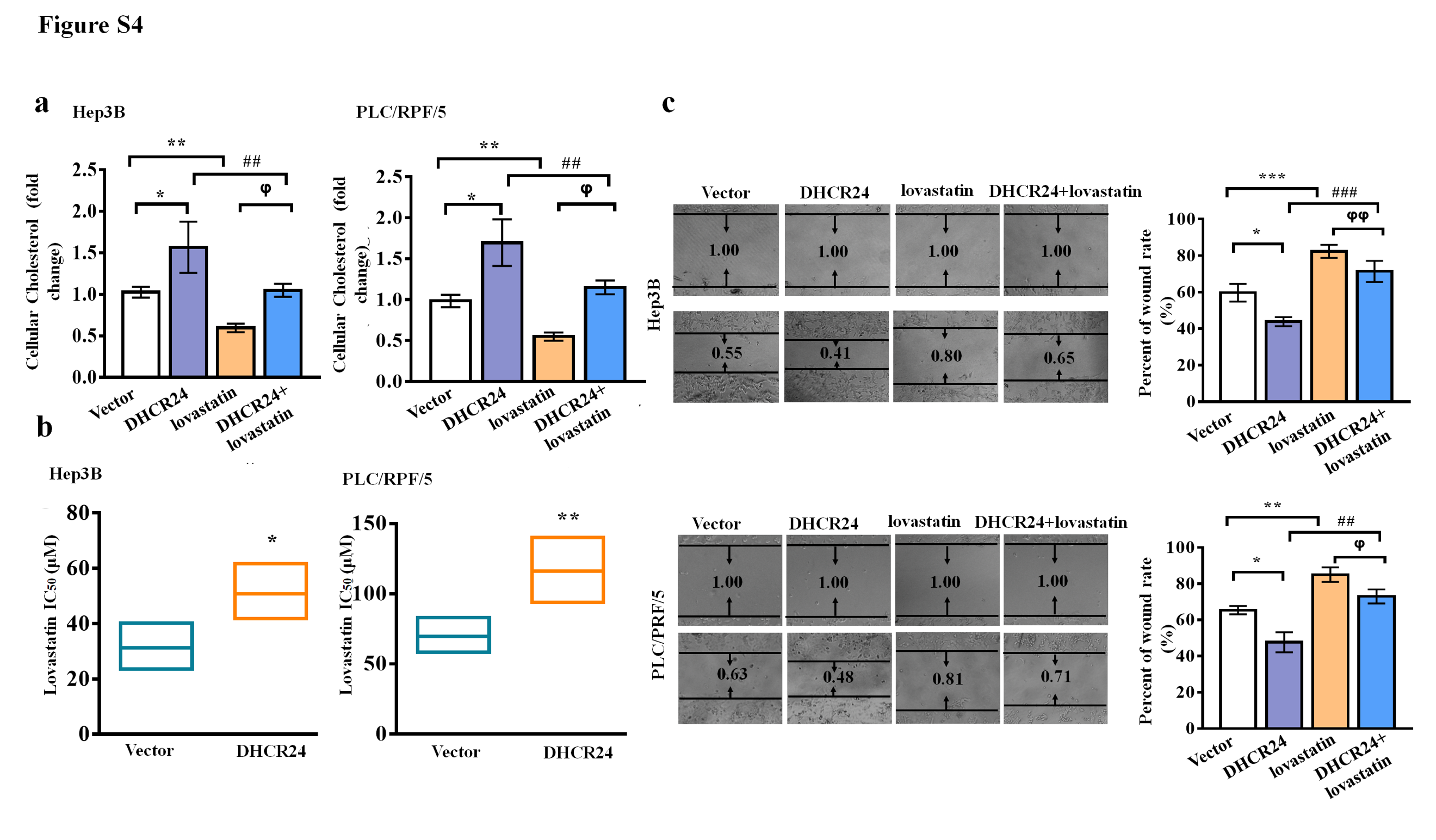
**

**
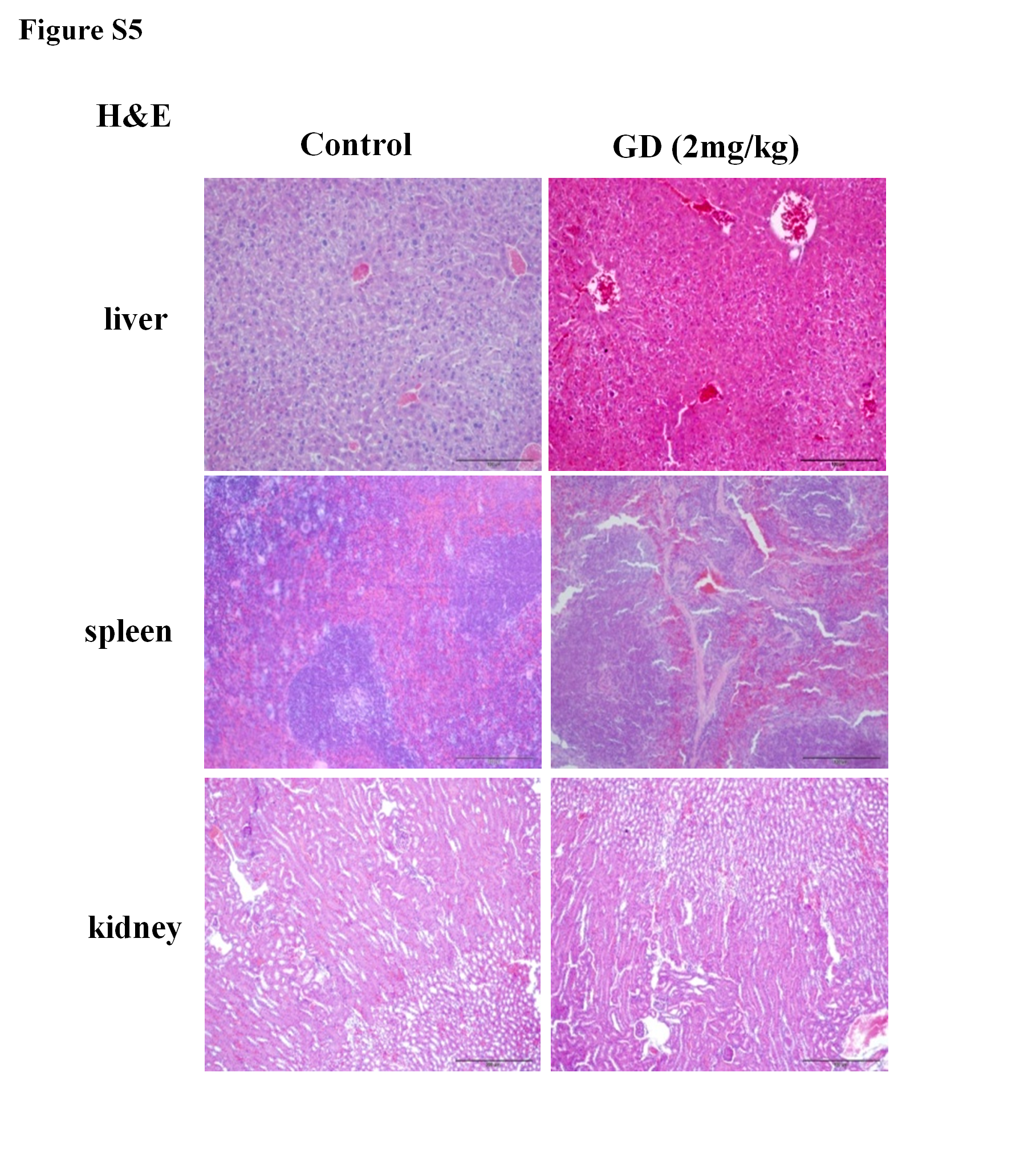
**

**
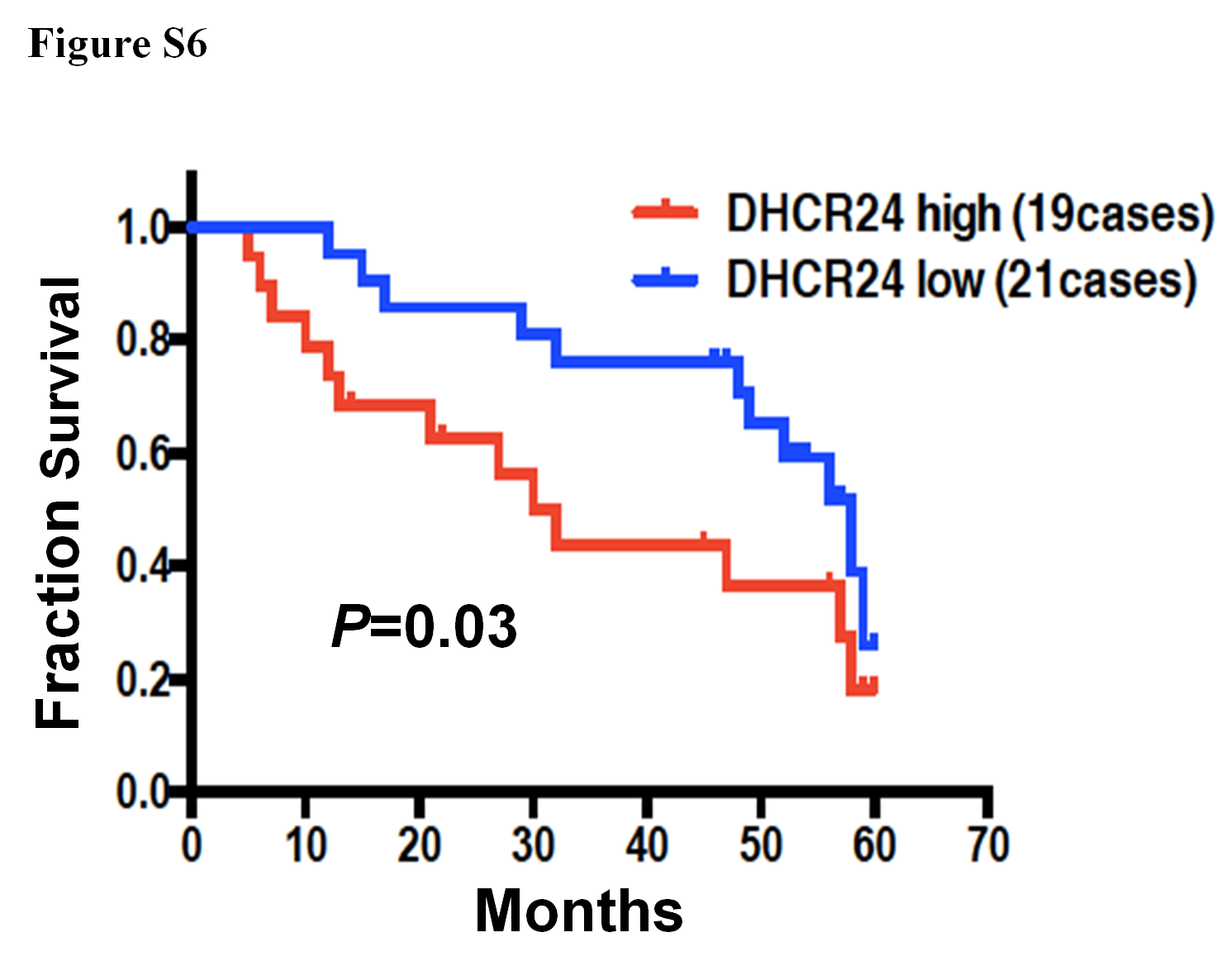
**

**
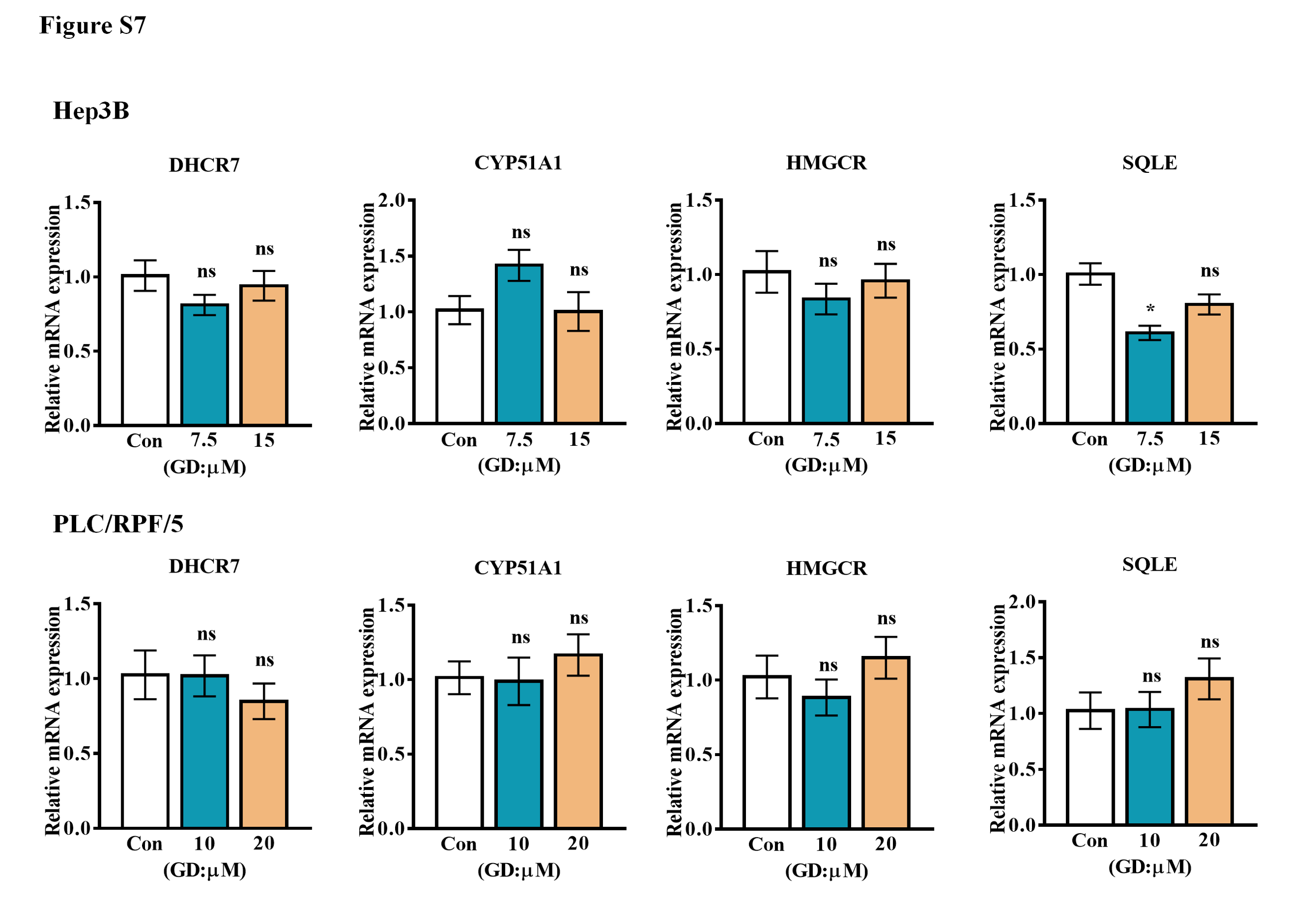
**
